# Challenges and support systems of nurses caring for women with advanced cervical cancer in Accra, Ghana

**DOI:** 10.1186/s12904-024-01507-2

**Published:** 2024-07-16

**Authors:** Jennifer Oware, Merri Iddrisu, Kennedy Dodam Konlan, Gladys Dzansi

**Affiliations:** Department of Adult Health, School of Nursing and Midwifery, University of Ghana, Legon, Greater Accra Region Ghana

**Keywords:** Cervical Cancer, Challenges, Ghana, Nurses and midwives, Resilience, Support systems

## Abstract

**Introduction:**

Cervical cancer is one of the causes of female deaths worldwide. Cervical cancer incidence is rising with almost three thousand (2797) women in Ghana being diagnosed with the condition each year, with almost two thousand (1,699) of them dying from its complications Nurses caring for cervical cancer patients are exposed to emotional and psychological distress due to late presentation, the burden of care, patients’ suffering and the alarming number of deaths associated with it. Improving positive patient outcomes require identifying the challenges and support systems available to nursing staff so as to harness these support systems for improving care outcomes.

**Aim:**

This study explored the challenges and support systems of nurses caring for women with advanced cervical cancer in Accra, Ghana.

**Method:**

In this study, we adopted an exploratory qualitative design. The study was conducted among eleven (11) nurses and nine (9) midwives engaged at the national referral hospital in Ghana who were providing care for patients with advanced cervical cancer for over a year who were purposively sampled. The data was collected using in-depth interviews with a pre-tested semi-structure interview guide from the twenty participants. We recorded the interviews using an audio-tape. The audio files were transcribed verbatim and thematic analysis was undertaken with the aid of Nvivo 10.0.

**Results:**

The challenges when rendering nursing care faced by participants of this study were exposure to frequent deaths, inadequate resources, and workload. Most participants lamented that they received absolutely no support from their workplace, hence their only form of support was from their family and friends. They also added that most of them were general nurses and midwives with no special training in oncology nursing or palliative nursing.

**Conclusion:**

Nurses and midwives experience resource, knowledge and skill challenges when caring for patients with advanced cervical cancer. However, the nurses and midwives had emotional attachment to their jobs and their patients and were not distracted by their bad experiences. We recommend improving resource allocation for cervical cancer care through the National Health Insurance Authority (NHIA), Ghana and increased training of nurses in oncology and palliative nursing by the Ministry of Health, Ghana to improve knowledge and skills of the nurses and midwives caring for women with advanced cervical cancer to improve their quality of care. Further, hospitals must make it a priority to have more nurses and midwives trained in oncology and end of life care to improve the knowledge and skills of nurses and midwives caring for advanced cervical cancer patients. Also, these findings should trigger policy-level discussions at the Ministry of Health, Ghana on the training of specialized nurses and midwives in cancer and end of life care to help Ghana meet the sustainable development goal targets related to health.

## Introduction

Cervical cancer has been identified as one of the main cause of morbidity and mortality among women worldwide [1]. Majority of the cases and death estimated in 2020 were recorded in low- and middle-income countries (LMIC) such as Kenya, Carribean and Malawi, where most affected women report to the hospital in a very debilitating state resulting in the need for palliative care, making cancer care more demanding and stressful [2–4]. In Ghana cervical cancer has been ranked as the leading cause of cancer related deaths among women [5]. According to Nartey and colleagues [6] state that an estimated age standardized incidence rate and mortality rate of 29.3/100 000 and 23.8/100 000 occurs annually in Ghana. Several studies have indicated that patients living with cervical cancer and all other cancers report to the health facilities at very critical stages making management problematic [4–7].

Cervical cancer incidence is rising with almost three thousand (2797) women in Ghana being diagnosed with the condition each year [5, 6], with almost two thousand (1,699) of them dying from its complications [1, 4–6]. Unfortunately, most cases present with advanced disease state and this places a heavy strain on nursing personnel who work in oncology settings [3, 5, 7]. This is attributed to a lack of knowledge, stigma, and misconceptions surrounding cervical cancer and its treatment [6, 8], thereby making cervical cancer care difficult and demanding. Additionally, nursing personnel in oncology settings in Ghana may encounter additional workplace stresses such as a lack of staff and inadequate resources, which may deter their enthusiasm in the field [7, 9]. Moreover, working in an oncology setting is demanding spanning from diagnosis to treatment including assisting with surgical interventions, radiation therapy and chemotherapy medications coupled with side effects management, symptom management, as well as palliative care at advanced stages of the disease [4, 10]. These expose oncology nurses to unique occupational hazards that are not common in other fields of nursing [11].

As the incidence and mortality rate of cervical cancer keeps increasing, the challenges with care also keep rising [12, 13]. Nurses working in oncology units are constantly exposed to patients’ suffering, pain, anxieties, death, and many others [14, 15]. Cancer care has been identified as a demanding and stressful area of practice in relation to the intensity of its management. Oncology health professionals work in stressful environments to meet the special needs of their patients and their families. In some instances, their health is compromised to save their patients [16, 17]. Workplace stress in the oncology field is a result of the unique emotional demands of the field, such as dealing with terminally ill patients, intricate cancer therapies, and the inevitable loss of life [16–18]. Nurses are the most affected among the multidisciplinary team of oncology management because of the non-stop care they offer to cancer patients especially those at the terminal stages [19]. The situation is worsened especially for new nurses who lack the requisite skills and experience in providing end-of-life care to patients and their families [16–20]. The literature on cervical cancer in Ghana [5, 6, 20, 21] has largely focused on the experiences of patients and family caregivers suffering from cervical cancer, knowledge and awareness on cervical cancer and screening and male involvement in cervical cancer screening but little is known about the challenges of nursing staff caring for women with advanced cervical cancer and the support systems available to these nurses and midwives. These challenges among Ghanaian health workers could be similar to those in other parts of the sub-region and hence the findings of this study would be useful in instituting interventions to improve cervical cancer care in most countries in sub-Saharan Africa and other parts of the world where the health systems are bedeviled with numerous challenges [6, 20].

## Aim

This study explored the challenges and support systems of nurses caring for women with advanced cervical cancer in Accra, Ghana.

### Conceptual framework underpinning the study

**Fig. 1:**
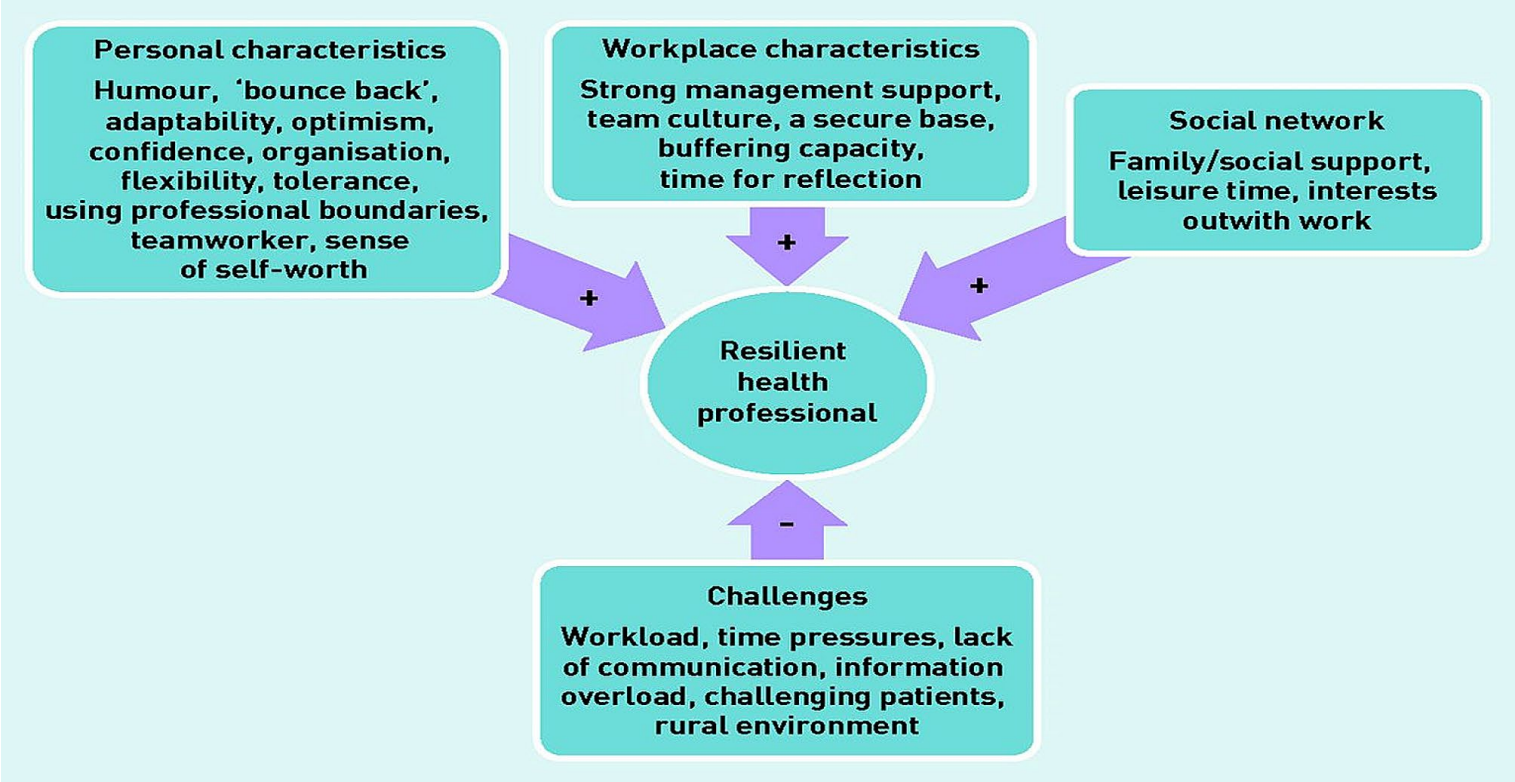
The model of health professionals’ resilience

This study was underpinned by the Health Professionals’ Resilience in Primary Care model. According to Matheson et al. [9, 21–24] the Model of Health Professionals’ Resilience in Primary Care was created to better understand what primary health professionals in high-pressure environments consider to be resilience qualities and what promotes or challenges professional resilience. The model is characterized by four main constructs namely personal characteristics, workplace characteristics social networks, and challenges. According to Matheson et al. [9], three constructs including personal characteristics, workplace characteristics, and support systems work in synergy to promote resilience while the last construct, challenges has a negative influence on resilience. The model of health professionals’ resilience was considered the most appropriate framework for this proposed study as its constructs fits the objective of the study. This model was preferred as the theoretical model for this study because its constructs aligned with the focus of the work. The constructs of the model are presented in Fig. 1.

## Methods

### Study design

We used an exploratory qualitative design [22]. This design was chosen to obtain detailed insights into the unique challenges and support systems experienced by nurses and midwives when rendering care to women with advanced cervical cancer. The exploratory qualitative design is useful for providing in-depth insights into a phenomenon of interest [22] and was thus appropriately selected for this study.

### Setting

The study was conducted at the national referral hospital in Ghana. The Hospital is called the Korle Bu Teaching Hospital (KBTH) located in Accra, Ghana. The Hospital has a cancer registry in which the number of cancer patients are recorded. Breast cancer was the most frequent malignancy encountered at the KBTH (80.5 cases per 100,000 hospital attendance), followed by cervical cancer (46.9 cases per 100,000), prostate cancer (21.8 cases per 100,000),

and colorectal cancers (13.8 cases per 100,000) [8]. Breast cancer accounted for 40.8% of all cases in women, followed by cervical cancer at 24.3% and uterine cancer at 4.5% [5, 6]. The hospital has seventeen departments and this study was conducted at the Gynecology Unit of the Obstetrics and Gynaecology Department. The data for the study was collected among nurses and midwives at the gynaecology unit of the KBTH. This unit was selected because it acted as a referral centre for complicated and advanced cervical cancer cases in Ghana.

### Study population and sample size determination

Nurses and midwives caring for patients with advanced-stage cervical cancer were targeted for the study. These were nurses attached to the gynecology unit of the Obstetrics and Gynecology Department of the KBTH. The participants were recruited if they met the following inclusion criteria: Those who had a current license as a registered nurse or midwife, those who had over a year professional nursing experience caring for cervical cancer patients and nurses and midwives who had given care to a patient with cervical cancer in the previous twelve (12) months.

We adopted the non-probability sampling technique of purposive sampling to select the nurses and midwives to participate in the data collection.

During the interviews, we noticed that redundancy started after the sixteenth (16th ) interview and data saturation was reached at the twentieth (20th ) interview. We added four additional respondents after redundancy started to reach data saturation, where no new information was emerging from the interviews. Hence, twenty (20) nurses and midwives took part in the study as there was no new information after the 20th interview and thus data saturation.

### Selection of participants and data collection

The Participants were selected from the Gynecology Unit of the Obstetrics and Gynecology Department of the KBTH on weekdays. We adopted a purposive sampling technique and recruited only nurses and midwives caring for women with advanced cervical cancer. Nurses and midwives were included in the study if they met the following criteria as stated earlier: registered nurses/midwives with over one year working experience caring for women with advanced cervical cancer and currently providing bed-side nursing/midwifery care for women with cervical cancer within the last one year. We excluded student nurses/midwives, unprofessional/auxiliary nurses/midwives and those with less than one year working experience caring for advanced cervical cancer patients.

The data collection was done between July, 2022 and September, 2022. We initially contacted about sixty-four (64) nurses and midwives working in the Gynecology Unit but only forty-eight (48) expressed interests in participating in the study. However, not all those who agreed to participate took part in the interviews as there was redundancy and data saturation after the 16th and 20th participant’s interviews respectively. We provided a participant information sheet which explained fully the purpose, benefits, risk of the study among other things to each potential participant who had met the inclusion criteria. We then obtained written informed voluntary consent from each participant who expressed desire to be part of the study.

The data was collected at the Nurses rest room attached to the gynecology Unit using a semi-structured interview guide which had five sections/parts. It contained open-ended questions that allowed further probing to elicit relevant responses about the experiences of the respondents in the care of patients with advanced stages of cervical cancer.

The first section of the instrument solicited data on the background information of the respondents. Data collected included the age, highest educational qualification, years of experience, marital status and religion, number of children, and number of dependents. The researcher believed that this information was relevant to determine the personal characteristics of the respondents and established rapport with the participants. Data to address the objectives of the study was gathered from the fourth and fifth sections of the pre-tested interview guide which focused on the challenges and support systems of the nurses and midwives as they rendered care to the patients with advanced cervical cancer. Some of the questions from the interview guide included: Tell me about challenges you encounter in your work? What has been your greatest failure in your course of work?, Do you think this has shaped you as a person?, What will you do differently next time? Share with me how your workplace challenges have affected your personal life? Tell me about any health challenges you have faced as a result of your work? Tell me about support systems that help you cope with the work? Tell me about the support systems at your workplace?, Discuss with me how you personally deal with stress? How do you keep your fellow co-workers upbeat at the workplace? Share with me how you think resilience can be developed among others. We asked the participants to further describe in detail their responses to the questions. This tool was pre-tested at the Komfo Anokye Teaching Hospital in Kumasi, Ghana and was found to elicit appropriate responses to answer the objectives of the study.

We used an audio-tape to record all the in-depth interviews with the participants. The interviews lasted 45–60 min for each participant. In addition, we maintained a field note book in which we recorded important incidences during the data collection as well as key observations. The recorded audio files were transcribed verbatim and thereafter thematic analysis was done.

### Quality control

To ensure the quality of the data collected, the semi-structured interview guide was pre-tested at the Komfo Anokye Teaching Hospital (KATH), the second largest teaching hospital in Ghana among a similar class of nurses and midwives caring for cervical cancer patients. The goal of the pre-testing was to assist to ensure clarity of the questions in the interview guide and also determine whether the questions sought to answer the research questions [22]. Questions that were observed to be narrow or capable of generating restrictive responses were modified to include the words “describe” or narrate to broaden the responses and to give the participants the opportunity to speak more on the topic.

During the data collection, the researchers adhered strictly to methodological rigour described below and also enhanced member checking with the participants were done to ensure that their views had been properly represented in the transcripts from the interviews [22]. At the end of the data collection, the audio transcripts in English were confirmed as verbatim transcripts of the audio files.

### Data management

We ensured that all the interviews were audio recorded and the data was kept in a folder with a password and accessible only to the researchers. We carefully transcribed verbatim all the recorded audio files from the interviews into text. We then compared the transcripts with the audio files several times to verify any discrepancies and contacted the participants where there was a need for clarification. We also ensured that the participants were given unique codes based on their wards and their position among the participants. Participants’ confidentiality was ensured by removing all identifying attributes from the data and replacing them with pseudonyms. All identifying attributes such as contact information were stored electronically in a password-protected laptop, likewise consent forms, field notes and other important documents were kept under lock and key and the files are only accessible to the researchers. The data was also stored on an external hard drive accessible to only the researchers to prevent data loss.

### Rigour

We adhered strictly to the tenets of methodological rigour required in qualitative studies [22]. To ensure credibility, the interview guide was pretested among two patients who met the inclusion criteria at the Komfo Anokye Teaching Hospital’s Oncology Department, Kumasi-Ghana. This allowed the researchers to make necessary modifications to aspects of the interview guide that seemed not to elicit responses that were relevant to the objective of the study. Additionally, a purposive sampling technique was employed to ensure only nurses and midwives caring for women diagnosed with advanced cervical cancer who could give a vivid account of their experiences were involved. Probing and iterative questioning was also used to elicit responses from participants and situations where there were ambiguities in the responses; clarifications were sought from the participants. To achieve transferability [22], the research process was described in detail so that others can evaluate the applicability of data to other contexts and settings. Records of the transcribed interviews and the analysis, as well as the results of the study, were kept for audit trail and are in the custody of the 1st author. To ensure dependability in the study, a semi-structured interview guide was used for all the interviews to ensure consistency in the line of questioning among the participants. Again, a detailed description of the study design, sampling method, data collection, and analyses were as well.

documented. To ensure confirmability, the context of data collection was documented in a field note book during the interviews. This enhanced interpretation of the data during analysis to reflect the exact responses of the participants. The authors also bracketed their experiences and presuppositions to avoid any influence in the interpretation and analysis of the data.

### Data analysis

We performed the data analysis concurrently with the data collection with the aid of Nvivo 10.0. The researchers adopted the thematic analysis approach to data analysis as recommended in literature [23, 24]. The interviews that were audio recorded were transcribed word for word as soon as they were concluded. The verbatim transcripts and the field notes recorded in the diary were used in the thematic analysis. The thematic analysis used in this study followed the procedure (six phases of data analysis) as outlined by Braun and Clarke [23, 24]. Thus, the thematic analysis that was carried out involved the following stages: familiarization with the data/transcripts by reading through multiple times, generating initial codes as the data has been read, searching for themes, reviewing themes, defining and naming themes and producing the report. To begin describing the challenges and support systems for the nurses and midwives were grouped into meaningful units to generate the initial codes that led to the development of themes and sub-themes. In generating the codes, we read the data several times and as the data was being read, interesting ideas in the data were highlighted as codes that capture meanings from the narration of the experiences of respondents. We reduced the long paragraphs of the narration into meaningful chunks. The generation of the codes was theory-driven based on the theoretical model underpinning the study. We analyzed the data based on the theoretical model of Health Professionals’ Resilience in Primary Care by Matheson et al. [22–24]. The model is characterized by four main constructs namely personal characteristics, workplace characteristics social networks, and challenges and these formed the basis for the thematic analysis. We then sorted the different codes into potential themes and this involved sorting the different codes into potential themes. The naming of the themes was based on the constructs of the theoretical model used for the study. Direct quotations were captured in the analysis to support the themes generated from the transcripts.

## Results

The study had twenty (20) participants consisting of eleven (11) nurses and nine (9).midwives. The participants were nurses and midwives who had rendered nursing care to women with advanced cervical cancer for more than one year.

The data analysis yielded two (2) themes and eight (8) sub-themes. Table 1 presents the synthesis of the themes and sub-themes gleaned from the data (Table 1).

**Table 1:** Synthesis of themes and sub-themes

Major theme	Sub-theme
Challenges with care	Inadequate Human Resource
	Inadequate Material resources
	Unappreciative Superiors
	Unsupportive Leadership
	Favoritism from superiors
Support systems	Support from Colleagues
	Family Support
	Institutional Support

### Challenges with care

In this study, three key sub-themes emerged. They were; Inadequate Resources (inadequate human resources and inadequate material resources), Leadership/Managerial challenges (unappreciative, unsupportive and favouritism) and Workload (type of patient, demands of care, patient’s preferences, patient’s family neglect and delays).

The findings revealed that most nurses faced difficulties and frequently turned to alternative sources for help.

### Inadequate resources

Like every institution, the health sector needs resources to operate efficiently, whether they are human, material, or environmental. Participants in this study admitted that they have many resource-related difficulties. This sub-theme gave rise to the following two minor sub-subthemes: Inadequate human resources and inadequate material resources.

### Inadequate human resource

One of the ongoing issues in nursing care is inadequate staff to work on the ward. Eleven (11) out of the sixteen (16) nurses and midwives attending to patients with advanced-stage cervical cancer brought this issue to light in the following ways;

*“You come to work and you are just like 4 and you are taking care of 25 patients or 30 patients*,* it is the workload*,* is too much so we have issues with that…”.* (CNM06)

*“As for the challenges*,* there are many of them. As a result*,* our staffing levels are always inadequate. Looking at our nurse-patient ratio is woefully inadequate”*.(CNM13).

*“we are not enough as nurses and midwives to care for the numerous patients who come to this unit*,* its not easy” (CNM 15)*.

*“Here*,* the work is too much and the staff are very few” (CNM 11)*.

Also, some participants expressed that the issue with the human resource is not that they are understaffed but there are shortages occasionally due to staff gender.

“*Given that nursing is a profession where women predominate*,* most of the time*,* we get nurses going on maternity leave*,* some are pregnant and all that which adds up to the staff shortage*” (CNM16).

“Some of our colleagues are on maternity and annual leave, so we are few at post and this puts pressure, unnecessary pressure on us and no one cares” (CNM 03).

“Our staff in this unit are few because some are on leave and is like the leave is not coordinated at all” (CNM 09).

Again, other participants expressed that the staff shortage they have is due to the stressful nature of their work which causes some staff to experience burnout and others to report sick, causing them to bring excuse duties which worsens the issue of staff shortage:

“*Also*,* the stress here is unbearable*,* so we have most of our staff bring in excuse duties*,* reporting sick and all that so for staffing I will say is inadequate. For the personnel element*,* that is what I can say*”. (CNM15)

“my colleague that I was working with is now on sick leave and this has left me with pressure ” (CNM 04).

However, there was one respondent who had the opinion that, staffing is better now compared to the past;

“*With staffing*,* it is better*,* now it’s better. They posted some nurses here so it is better. At first*,* it was bad; you would come and you would be assigned to 12 patients. Am telling you. And when you come if it’s in the morning some would go for x-rays*,* go here…hmm…and the whole day*”. (CNM01)

“Even though the staff are many now, so are the patients and this puts pressure on the staff” (CNM 14).

### Inadequate material resources

When there is a lack of resources, it proves as an impediment that will lead to compromising the quality of care given to patients by nurses and midwives. These challenges were not only limited to human resource issues, but logistics to facilitate health care delivery were also lacking. There were the experiences by fourteen (14) nurses and midwives as stated below;

“*I like this*,* where you have gotten to because it is a big challenge. A challenge that I don’t expect to see from a very big hospital like [Mention the name of a Facility] but sometimes you come to work and the things to work with is a problem*,* things like…. logistics like syringes*,* BP apparatus*,* cannulas*,* giving sets. I don’t get it. I mean*,* the biggest referral centre in the whole Ghana and even a referral centre in West Africa*,* why*,* must such a hospital go through some of these challenges*”. (CNM03)

Other participants affirmed that to meet patient’s care needs, they had to improvise with what they have due to inadequate material resources.

“*Again*,* the logistics we use in working are frequently unavailable or insufficient*,* so we are constantly instructed to either improvise or to do our best with the few things we do have. At times it is so bad that we go around looking for items from other wards which I think a facility like ours should not be having*”(CNM16).

“You come to work with no logistics or materials to work with and this is a main challenge” (CNM 11).

“The bosses don’t care about us and they don’t provide the things we need to work, unfortunately most of the patients are poor and cannot buytthe things too” (CNM 07).

Worth to note that participants borrowed from other wards to meet the health needs of patients;

“*I will say in terms of logistics too is not enough*,* sometimes common gloves we have to borrow from other wards which is a big challenge because the work is already tiring*”. (CNM 09)

“Sometimes we go to the nearby wards/units to borrow things for work” (CNM 11).

Participants stated that the majority of patients receiving treatment are oxygen dependent, and that causes frequent oxygen shortages in the ward, which slows down work activities related to managing these patients. They cited the lack of oxygen as a major obstacle;

“*Oxygen is a big problem*,* most of our patients have breathing issues and depend on oxygen*,* but at times you will see nurses carrying oxygen cylinders from other wards just to save lives*”. (CNM11)

*“I remember sometimes on the ward three people are on oxygen and then another person needs oxygen you have to be monitoring the SPO2 too. So*,* let’s say this person’s SPO2 is a bit okay. Then we swap to save lives”* (CNM12).

Some participants also discussed how the lack of material resources has an impact on the level of care they provide, which is emotionally draining;

*“Again there are days when consumables to work with are not available*,* you try your best as a nurse manager*,* you go to all the things that you can go to and yet still you don’t have the consumables to work with*,* it makes you emotional*,* it makes you sad because you just ask yourself why they cannot get you the consumables you need to work with because at the end of the day infection prevention measures must be used and if you are not providing us with the basic requirements like gloves. Hmmm*,* it disturbs me a lot. You realize that the staffs are not happy everybody looks angered*,* they are not happy at all and some would be sad but this is where we find ourselves. So*,* they all come to you as the ward in charge and you also look sad and angry at the same time because they expect that you provide them with the items and where you are supposed to get the items too you are not getting”.* (CNM08)

Similarly, some participants expressed that the beds on the ward are in deplorable states making caregiving very difficult. Below is what they had to say:

“*Again*,* for now*,* a major challenge for me on the ward is also the state of the ward especially the state of our beds in the ward are in a deplorable state and anytime we draw the attention of the superiors they say is in the pipeline like trying to get new ones not knowing when it would come in.*,* we have patient falling*,* patient complaining*,* we have some of the beds even not for patients to be lying on*,* that is what we have as at now*”. (CNM16)

### Lack of a conducive environment

The quantity of hours spent at work by nurses and midwives is relatively high. The attitude of personnel toward work is influenced by the environment, which is perceived by the participants as unhealthy or unconducive.

Some participants complained of torn window nets which give way to mosquitoes leading to patients and staff getting malaria:

“*…the ward environment itself brings so much stress. For example*,* in my unit*,* all the nets are torn*,* and the nets cover the window. And at night the patient and staff have to battle with mosquitoes…. yes! and almost every week one is struck down with malaria…this is a lot of stress to us because at the end of it would affect my staff’s strength…. So*,* the ward environment is a big problem for me*”. (CNM08)

Some participants lamented that there was too much heat on the ward making them uncomfortable whilst at work:

“*…the environment is not encouraging because there is a lot of heat in the ward*,* so sometimes when you are working you would be sweating and you won’t feel comfortable to even work”.* (CNM02)

*Poor ventilation the ward is very hot sometimes by the time you finish working then your entire uniform is soaked in sweat. The washrooms too are not neat*”. (CNM07)

Similarly, some respondents also revealed that the signs and symptoms of cervical cancer at the advanced stages bring about some unpleasant odour which affects caregiving.

*“And sometimes because of the cancer patient has some scent*,* so sometimes the ward will be smelling like faeces*,* discharges and so many things*,* so the ward is not encouraging*,* scent. So sometimes because of the scent around a patient*,* when you are working on a patient you are in a hurry to leave because of the smell which is not encouraging and the ventilation is very poor*”. (CNM02)

Again, some respondents termed their working environment as discouraging due to the absence of a medium of entertainment such as television and radio as a source of diversional therapy both for patients and staff:

“*…sometimes before we take up*,* we are already tired and the environment too is very discouraging. If you look at the ward there is no tv. Sometimes the patient might feel exhausted at the same time experiencing pain. If there was a television here it could take their mind off the pain. Hmmm*,* we are stressed. Am not saying we should come and play music at work but something to at least make the patients happy and expand their lives. No television to entertain us*,”. (CNM07)

Again, some participants expressed that, the unkempt nature of the washrooms on the ward made the environment unconducive which was a challenge to them:

“*Sometimes the washrooms because of the bleeding all the time. The place is not well kept some of them don’t keep the place neat and then all the time you have to be getting people and some the orderly’s coming they’re angry. somebody will remove the pad puts in the WC*”. (CNM12)

### Leadership/Managerial challenges

Nurses and midwives in this study expressed that, some superiors don’t appreciate their efforts and jump to conclusions whenever patients and relatives report them which is a challenge for them;

“*Yeah*,* my challenge with the superiors is that*,* as I said earlier*,* some of the superiors don’t appreciate what we do and even when the patients’ relatives report the nurses to them*,* instead of them listening to our side of the story*,* they won’t and they would just attack you like that. They always say the patient is right”. (*CNM02)

Some participants expressed that they are misunderstood by their superiors;

“*Our superiors too tend to misunderstand us anytime a problem comes up*,* they don’t listen to our part of the story*,* they just jump to conclusion*,* which at times is very painful*”. (CNM10)

Similarly, some participants also lamented the fact that some in-charges when on duty are found ‘table nursing’ instead of supporting them which increases their workload;

“*Hmm! Some of the in-charges when you come with them*,* do table nursing*,* they would be sitting by the table writing. At times too not writing but they would be ordering you around. There are instances that the in charge has to see the patient but they would be sitting down*,* then sharing patients but they wouldn’t know what is going on in the wards. That too is a problem. The table nursing is a problem*”. (CNM01)

Again, some participants complained of delayed information by superiors when colleagues report to work sick or seek for excuse duty leading to staff shortages with increased stress and workload;

*“Also*,* with my matron for instance*,* when a colleague reports to her that he or she is sick*,* instead of her informing us the nurses on duty that may be your colleague asked for permission she won’t and when we come and we share the work we leave her portion there until later when you call that particular nurse before she will tell you that am sorry am not well*,* I reported to matron so am not coming to work”.* (CNM16)

Additionally, some participants had the opinion some superiors had their favourite staff which affected relationships with some of them;

*“There is also favouritism*,* especially in my ward. My matron likes some people more than others because some people will not even bring excuse duty but they will be granted permission. Someone can just stay home and call that my father is sick and so she wants to take care of him and will be granted permission*”. (CNM19)

Some nurses and midwives also expressed that, looking at the stressful nature of their work, patient’s family support would be helpful, but they are being stopped by superiors when the opportunity presents itself;

“…*And so*,* if the patient’s relatives are ready to come and assist the nurses and the superiors will come and say that it’s not their job*,* it’s the job of the nurses to do and other things. It means they don’t have us at heart*,* they don’t have us at heart do you understand yeah? So then again it leaves us with a lot of conditions*”. (CNM20)

Moreso, some respondents had the view that if superiors could have listening ears for them and confidentiality it would have been helpful;

“*Hmm! I think one*,* for me if we had open leaders or leaders who are willing to listen to us when we have problems*,* they always say that if you have a problem you can come to us but you talk to a senior issue about your problems and the next minute more than 3 or 4 people are aware of it. So*,* whenever you have a problem*,* you rather prefer to keep it to yourself”*. (CNM08)

However, few of the participants feel superiors are trying their best in terms of support;

“*Hmmm…. Our matron is our greatest support*,* she understands how the work is so she is always behind us*,* she is always defending us. So*,* I think…. Hmmm”*. (CNM05)

### Workload and burnout

Some participants revealed that the magnitude of the workload is too much causing staff to report sick and request for excuse duties which further increases the workload of the few available staff;

“*Okay*,* personally when staff comes with complaints*,* and*,* you are with them*,* I feel it puts pressure on other staff. So*,* if people keep on bringing up excuses because they are stressed*,* then all the workload will be on a few people who are not complaining. So*,* let’s say you come to work*,* you’re supposed to be three. And now it’s only two people coming or one person coming*”. (CNM12)

Similarly, some participants expressed that their workload is intensified due to the type of patients they care for, who are most critically ill and bedridden;

“*The number of patients coming in! and the level of help we have to give them. Most of them at the terminal stage*,* become bedridden*,* and they come in with DVT that they won’t be able to walk by themselves. You have to carry them or wheel them to the washroom or if they are going to take scans or x-rays*,* they can’t walk by themselves so you have to put them in a wheelchair if the patient is heavy or not heavy you have to. That makes the work…that part of it makes the work very stressful*. *Okay so*,* physically it is very stressful*,* especially when the patient becomes immobile like the patient is bedridden and all that. Yeah*,* it’s a bit stressful to handle such a patient*”. (CNM05)

Additionally, the study discovered lifting as a major stressor of nurses and midwives managing patients with advanced-stage of cervical cancer because most of their patients are bedridden;

“*The major stressors are the lifting. Because the patient cannot do anything for themselves. Some are bedridden so you have to lift them. Even when you are changing their diapers and soiled linen you have to raise them so sometimes you have to employ correct body mechanics and even with that the bending still exits. So sometimes you get home with waist pains*,* you will be tired having pain all over your body*”. (CNM14)

Moreso, the study revealed that the demand for care is intensified as a patient’s condition advances;

“*Like I said they come in when the condition is deteriorating and so most of them are bedridden*,* have respiratory problems and all that. So basically*,* we do everything for them which adds to our workload. You get home so tired to the extent that you don’t have the energy to do anything else. Sometimes*,* I don’t even eat nor bath*,* I just sit on my sofa and am gone*”. (CNM11)

Also, some participants also revealed that patient preferences are contributing factors to participant’s stress and workload;

“*Even some of the patients if you bathe them in bed will not agree*,* you have to take them to the bathroom and you have to stay with them even if they have to empty their bowels because if you leave them*,* they might fall and die. Recently an incident like that happened by the time the nurse got there she was dead*”. (CNM07)

Again, some participants expressed that, sometimes their competency is questioned by patients when the condition advances which becomes unbearable for them;

“*For example*,* when they get to the terminal stages*,* rejected by family and all that. At times you go to administer a drug to a patient and they even doubt your competence. They feel you want to help end their lives because they are stressing you*”. (CNM09)

Similarly, participants lamented that, when patients get to the advanced stages of cervical cancer, they even doubt the medications served:

“*Sometimes*,* especially those who have been rejected by their family often feel that the nurses want to kill them when they are been served with their medications*”. (CNM07)

“*I once had an encounter with a patient who when it got to the end stages asked me to take the drug first while I was serving medication because he didn’t trust the nurses anymore. They also become addicted to some of the pain medications and once we don’t keep the drugs and the drugs are with them*,* sometimes by the time you go to them they have already taken the drug*,* when you complain they become angry*”. (CNM11)

Additionally, participants revealed that their workload and stress are heightened when patient’s family members abandon them leaving nurses and midwives to perform the roles of relatives;

“*Some of them too their relative will come and damp them here and then will not even buy the prescribed drug for them which at times we have to contribute and do so. We have discharged patients; some have been here for three months now while they’ve been discharged*”. (CNM01)

To add to, participants revealed that not only does the patient’s family neglect increase their workload but it also burdens them financially;

“*Some of the relatives are not helping at all*,* they bring the patients here and you don’t see them again*,* so you have to do everything for the patients. Is like the relatives are waiting for the patient to die so that they come for the body. At times we have to organize and buy certain things for the patient*,* it is very heartbreaking*. (CNM13)

However, some participants revealed that is not just about the workload in caregiving but some external factors adding up to their stress;

“*As for [mentioned a name of a facility] stress*,* can you? you get home very tired. Even now am pregnant*,* I get to work and am assigned to about four patients to care for them*,* and I have to do everything for them so I get home very tired. Sometimes*,* when you get home*,* you can’t bathe by the time you realize*,* the next day has come*”. (CNM07)

Participants of this study revealed that most of their patients only seek care when the condition has advanced making caregiving problematic;

“*And they mostly come when it’s stage three B’s*,* stage four. I mean*,* the stages where cancer has gotten to and there is nothing much the doctors can do or the health team can do to make sure this patient comes out of that situation. And that makes it more stressful. So*,* the psychological*,* should I say trauma*,* or should I say stress*,* or imbalance*,* so we go through all of that*”. (CNM03)

The majority of participants expressed that they are exposed to frequent deaths of their patients because most of them seek care when the condition has advanced. They expressed this in the following ways;

“*Here the deaths are a lot compared to maternity*,* most of the cases that are being bought here are in advanced stages so the deaths are a lot. Most of the patients wait until the cancer metastasis to either their liver or kidneys…before they are brought to the hospital.* (CNM16)

Participants revealed that the reasons why patients report to the hospital in advanced stages are because most of the patients seek spiritual help and delay in churches;

*Some when you interview them will tell you that they were in the church for a while and seeing the condition was getting worse*,* they were asked to go to the hospital and were referred here at the [Mention the name of a Facility] teaching hospital. So*,* by the time they get here*,* the condition has become worse. They would have deteriorated and then nothing could be done about it. So*,* if we don’t take care …. then they go off*”. (CNM01)

However, it was revealed that the delay in seeking care by cervical cancer patients is due to inadequate information on cervical cancer

“*Cervical Cancer patients come in the wards most times err as you’re seeing terminally ill because they don’t have prior information about their condition at the time they come*,* they are almost at their terminal stage*”. (CNM12)

### Support

This sub-theme seeks to answer the question “what are the available support systems for nurses and midwives in cervical cancer care?”. From this sub-theme, the following headings were discussed; support from colleagues, patient family support, participant’s family support and institutional support. All participants expressed their views on this.

Some participants revealed that support from colleagues serves as a source of support that helps them bounce back from stressful events;

“*What helps us is that*,* when we come to work*,* we entertain ourselves because we know that no matter what we have to do the work*,* so the staff-to-staff relationship helps*” (CNM07).

“*Is the teamwork here that also keeps us going. So at least we have each other’s support*”. (CNM13)

### Patient’s family support

Participants revealed that patient’s family support is inadequate, in that most relatives dump the patients on the ward without visiting making caregiving difficult;

“*Like I said when you come for night duty*,* you’re the only person taking care of a bed-ridden patient you understand*,* and like I was saying if we get assistance from the patient’s relatives*,* especially the bed-ridden ones*,* it will make our work less difficult. But here is the case whereby the patient’s relatives are ready to come and assist us and then our superiors will come and tell them that they don’t need like they shouldn’t come and assist us and we are the ones supposed to do like literally everything*”. (CNM06)

### Participant’s family support

It was revealed from this study that the major support system for participants is their families, which they mentioned; parents, spouses, children and cousins:

Some expressed they get their support from extended families such as cousins:

“*You can’t do that alone. Hmmm*,* me like this I have a cousin who helps me with the kids*,* other than that it won’t be easy at all. But those without helpers*,* if they come in the morning and you see their faces…hmmm…. but me at least I have someone who helps me. When I get home and am tired*,* she helps does whatever that is supposed to be done* and *if only your husband understands there is no problem. At least you have your 3 days off…yeah so if only they would understand*,* there would be no problem*”. (CNM01)

Some revealed their mothers as their support system:

“*Yes*,* I have my mother’s support. She is with the kids now and is here. That is the greatest support I have. For my colleagues*,* we are co-working together. The environment is conducive though it is tedious because we support one another it becomes less stressful and conducive to work*”. (CNM05)

Similarly, some participants expressed how relieved they become seeing their children after a day’s work:

“*Yes*,* I would say my support is from my family*,* because no matter how sad I am no matter how angry I may be when I get home*,* once the children see it*,* I mean they know me so well*,* in fact*,* the whole family they know me very well so once they see my facial expression*,* they know that something is wrong so they would surely ask about it then I won’t look down*,* so I think for now they are my support system. They are my strength*,* the times I come home distressed and tired*,* they are my strength because of their words of encouragement*,* my children would say*,* mommy*,* don’t worry everything would be fine*”. (CNM08)

The majority of respondents had support from their spouses which is so encouraging;

“*My husband has been of great support to me*,* he understands my work and I think that contributes a lot because at times you get home late and even for you to cook is a problem…*”. (CNM13)

However, some participants revealed that the nature of their job is on a verge of breaking their homes;

“*It is not easy because right now I come for the afternoon shift when I close by the time*,* I get home late*,* I am tired and want to sleep and when I cook my husband doesn’t even eat because is late and on my off days too*,* I have a lot of things to do. I don’t have time for the family at all because am always tired. No time for communication. And the food is the problem. What the family would eat. So*,* marriage and work are not easy. And because these patients are dependent*,* you get home so tired*”. (CNM07)

### Institutional support

To implement health promotion, illness prevention, and health protection activities effectively, institutional support entails making sure that there are efficient organizational structures and mechanisms in place. Most healthcare personnel lack specific training in oncology.

Some participants lamented that there are no support systems for them in any way;

“*As for the facility*,* there are no support systems*,* I don’t know if they are now doing something about it but there’s no support system…*”. (CNM01)

“*Hahaha*,* we don’t get any motivation from the hospital nor the superiors at the end of you will just work they won’t even appreciate you*” (CNM02).

Other participants had the opinion that management compounds their stress rather than supporting them;

“*At first this challenge was hitting me*,* but with time*,* the unfortunate aspect of it is our management too are not helping*,* you come to work and they will even compound your stress for you. Why didn’t you do this? Why didn’t you do that? And your whole mind will… If you don’t take care*,* you go crazy. Because already you’ve gone through… Let me say you came for the night*,* you’ve gone through a whole lot during the night*,* and in the morning your superior will come and be compounding the stress for you*” (CNM03).

Again, some described the support received from the facility as woefully inadequate;

“*The motivation is not the best. Yeah*,* so if we say that we are looking at the motivation am not sure we would come to work. And even now the salary is not even anything to write home about. And…. so those things would rather pull down the energy you are coming to work with*”. (CNM05)

Worth noting, participants lamented that they pay for seeking health care in the facility they work in, even though their illness resulted from their nature of work;

“…W*hen you’re sick too*,* they don’t do anything about it*,* usually when it started or it’s something that happened on the ward*,* the hospital doesn’t do anything about it. You take care of yourself*”. (CNM06)

Moreso, participants expressed that if not for the strict rule on transfer in their facility, they would have left;

“*But this is a situation where the condition of service is poor. You work with no motivation. Sometimes you could realize that if people work here to find another job elsewhere*,* they will leave. We are working in a health facility with no insurance knowing that you could contract an infection from a needle pick. By doing so they leave you to go home and cater for yourself expecting you to resume work thereafter. Don’t you think if such a person finds a better job in a better environment she will stay*,* the facility would have been empty if not for their strict rules on transfer*”? (CNM10) .

“I wish I can leave and go to another department, I am tired of this ward” (CNM 04).

“sometimes you feel you are BURNT with the work and you want a change of environment but that too is difficult, I wish I can leave this unit” (CNM 10).

## Discussion

A vital challenge from the findings was that there was a shortage in regards to staff who were attending to the care needs of these patients according to more than half of the nurses and midwives engaged in the study. When examining the issues facing the health sector in most developing nations like Ghana, human resource and logistical constraints take precedence [20]. This is one of the biggest problems in Ghana that prevents nurses from succeeding at their jobs. This is consistent with other studies [20, 25]. This increased stress level concerning duty performance and led to participants taking excuses thereby, reducing the staff strength further. The nature of care provided to these patients who were in the advanced stage of cervical cancer was comprehensive and more dependent since most were unable to do anything for themselves yet there are inadequate nursing staff. With such delicate patient needs to be met, the workload of the nurses and midwives will automatically be more packed and stressful. Nwozichi and colleagues [26], indicate that oncology nurses in Nigeria contend with limited resources and equipment and a shortage of qualified staff. This finding is not limited to Nigeria but similar situations exist in developing countries including Ghana [27] and other LMICs including Kenya, Zambia and Egypt [7] (Oti et al., 2021). In concordance with the literature [6, 8, 10], low staffing especially of nurses was associated with poor high mortality and vice versa of patients who were in the hospital.

The study found that common logistics that were used routinely for care were lacking or not adequate which impaired their performance. There were occasions when participants claimed they had to go to other wards with the hope of accessing some items such as syringes, gloves, oxygen and the like all in with the hope of saving their patients’ lives. They cited that this affected their ability to maintain infection prevention measures on the ward which was essential to both the participants and the patients in their care. They were also at times placed in a difficult position of deciding which patient had to be most prioritized with the limited supply of oxygen available despite having patients in the delicate condition who all must be supported in their breathing. To them, this should not have occurred since the facility was one of the teaching hospitals in the country. Similar concerns have been reported in other studies [9–11]. Resources for health care in most developing or emerging economies are poorly distributed if at all they exist [9, 11]. The participant further added these insufficiencies impacted the quality of care rendered to patients and put them at risk of work-related infections [8, 21]. Similarly, Nukpezah et al. [27], identified inadequate logistics as a challenge among nurses working in oncology settings.

The findings from the study showed that the environment in which the nurses and midwives worked was poor and discouraging not just to them but to the patients as well. Some of the nurses cited that there was no proper protection against mosquitoes on the ward since the nets were torn and no replacement has been done. This puts them at risk of contracting malaria which directly decreases the staff strength when any staff falls sick. There is also the problem of poor ventilation coupled with the odour of the ward because of the nature of the patient’s illness, the unkempt environment, and the lack of entertainment facilities. These made working in the ward uncomfortable and staff hurried to complete the task on patients so they could escape the heat and smell. The comfort of both staff and patients is critical and must be ensured. Besides, a discouraging environment will impact negatively staff output and quality care may be affected [8]. Similarly, other studies [12, 21, 27] suggests improvement in the work environment of health workers to reduce burnout and staff turnover. Perhaps nurse managers can solicit funds through religious organizations, non-governmental organizations interested in among others to aid in improving the environment where institutions have failed.

Unappreciative leadership was one of the findings from the study. Some of the participants were of the view that their leaders were unappreciative of their input towards the care of their patients since they made no time to listen to their part of the story when relatives reported them. However, a report from a relative that puts the nurses or midwives in a bad light was accepted as the truth and the nurse was reprimanded for it. Some leaders were also reported to limit their job description to working at the nurses’ station but refused to work by the patient’s bedside to assist other staff in attending to the care needs of the patients on the ward. Showing this kind of persistent feeling of not having staff at heart from the nurse managers will create a work environment that will prevent staff from giving their best because being shown praise for good work reinforces one to repeat the action [10. 11]. Therefore, nurse managers must balance their authority by giving praise where it is due and reprimanding where necessary to improve outcome levels [10]. Appreciative managers created a sense of value for the nurses and midwives [11].

Attending to cervical cancer patients’ care needs was acknowledged by the nurses and midwives as stressful. The deteriorating condition of the patients made meeting their needs more tasking since patients were unable to support their care personally. Having to nurse immobile patients involved a lot of physical strength from the nurses and midwives which had a physical effect on their bodies after work [11.27]. Despite employing appropriate body mechanics, the level of physical strength needed in these patients’ physical care was strenuous and crippling for the participants. Making it difficult and tiring to care for them.

Some of the nurses and midwives also added that some of the patients when in their last stage of the condition realizing there was no improvement in treatment begin to doubt their competencies. The participants noted that this behaviour was often witnessed among these patients who regard themselves as a burden to the staff and start being paranoid. As a test of trust in the participants, they revealed patients sometimes preferred nurses and midwives to take the medications intended for them first before they consent to take theirs. These attitudes can be futile in making positive impacts on patients’ treatments and giving comfort to the patient [14–17, 21]. The process of caring for a patient stem from recovery to comfortable death hence, in all the processes of care, the patient’s comfort must be assured [12, 21]. This can be done by appropriately involving psychologists in patient care and the nurses and midwives must also exhibit behaviours that are not regarded as hostile [11, 17]. A similar outcome was in Kim et al. [28] where the authors stated that cervical cancer patients in the advanced stage, lost trust in the care they were receiving as well as vital tests like laboratory investigations carried out.

Lack of family support for patients was evident from this study. This finding is similar to what pertains in the literature [25–28]. Apart from physical abandonment by some patients’ relatives, financial support was also an issue due to the expensive nature of treating cervical cancer. Financial assistance has been shown to have a significant role in helping cancer patients and nurses build resilience [27–30]. Such cases of neglect according to the participants, on occasions become a burden on the staff as they are left looking for ways to provide patients’ nursing needs in addition to soliciting funds to meet needs so that patients’ condition is not worsened. Furthermore, there is an indication from the study that this lack of support from family was pervasive as some patients upon discharge were left on the ward unable to leave for their homes since their bills were not paid. Even though there is the National Health Insurance Scheme (NHIS) Ghana, certain conditions are not absolved by this scheme including cervical cancer which must be looked at again. While some years past cervical cancer incidence was not high in the country, unfortunately, the story has changed now. It is therefore important that the NHIS takes some of the cost of treating cervical cancer into the scheme especially since the average Ghanaian will find it difficult to solely bear the cost involved in this condition.

The study findings pointed out that there was a lack of support from the institution. The study participants were of the view that the institution and the superiors were not motivating participants. Poor remuneration and lack of benefits especially for injuries or illness resulting from duty were some of the issues that made participants consider leaving for better pay and working conditions at other places. This finding is in line with other studies [8, 12, 21, 27]. When participants feel they are not being appreciated by the management and this acts as a catalyst for poor staff retention and resilience building [20, 28, 30]. This can be corrected by management instituting schemes that support career advancement [19, 29], and staff medi-care policies [20] among others could serve as incentives to participants. Specialized training of the nurses and midwives caring for cervical cancer patients in oncology and palliative care need to be pursued by the institutions that care for advanced cervical cancer patients.

### Limitations of the study

This study was conducted in a small fraction of nurses and midwives in the study setting due to the design of the study and the findings cannot be generalized. However, the in-depth data can be used as a basis for future studies. We recommend that future studies could evaluate this study area quantitatively using survey designs to enable generalization of the findings to the study population.

The study was restricted to the nurses and midwives at the gynecology unit of the Korle Bu Teaching Hospital so subsequent studies should focus on nurses and midwives in other facilities under the Ghana Health Service (GHS) since the conditions of service-delivery could be different due to different management strategies. We recommend for future comparative studies between nurses/midwives from the study setting and other study settings.

The findings of the study could have been affected by social desirability as the participants could have given responses to suit the social environment as with most questionnaire or interview-based studies. We recommend the addition of observation as a data collection approach in subsequent studies using the same design to elicit more objective study findings to improve workplace policies.

The findings only describe the phenomenon of interest and does not depict causation. We recommend for the use of more analytical and longitudinal designs in future studies to making the findings more robust.

## Conclusion

The study found several challenges when rendering nursing care faced by participants of this study which included exposure to frequent deaths, inadequate resources, and workload. Most participants lamented that they received absolutely no support from their workplace, hence their only form of support was from their family and friends. They also added that most of them were general nurses and midwives with no special training in oncology nursing or palliative nursing. This study revealed that nurses and midwives experience resource, knowledge and skill challenges when caring for women with advanced cervical cancer. However, the nurses and midwives had emotional attachment to their jobs and their patients and were not distracted by their bad experiences. The nurses and midwives worked-against odds to improve patient care amid the limited material, human, knowledge, and skill resources.

We recommend improving resource allocation for cervical cancer care through the National Health Insurance Authority (NHIA), Ghana and increased training of nurses in oncology and palliative nursing by the Ministry of Health, Ghana to improve knowledge and skills of the nurses and midwives caring for women with advanced cervical cancer to improve their quality of care. Health care institutions must make it a priority to have more nurses and midwives trained in oncology and end of life care to improve the knowledge and skills of nurses and midwives caring for advanced cervical cancer patients. These findings should trigger policy discussions at the Ministry of Health, Ghana on the training of specialized nurses and midwives in cancer and end of life care to help Ghana meet the sustainable development goal targets related to health.

## Data Availability

The datasets generated and/or analyzed during the current study are available from the corresponding author on reasonable request.

## Abbreviations

GHS: Ghana Health Service
KATH: Komfo Anokye Teachigng Hospital
KBTH: Korle Bu Teaching Hospital
